# Imaging episodic memory during development and childhood epilepsy

**DOI:** 10.1186/s11689-018-9255-8

**Published:** 2018-12-13

**Authors:** Leigh N. Sepeta, Madison M. Berl, William Davis Gaillard

**Affiliations:** 1 Center for Neuroscience Research, Children’s National Medical Center, 111 Michigan Avenue NW, Washington, D.C., 20010 USA; 2 Clinical Epilepsy Section, National Institutes for Neurological Disorders and Stroke, National Institutes of Health, 10 Center Drive, Bethesda, MD 20892 USA

## Abstract

Epilepsy affects 2.2 million adults in the USA, with 1 in 26 people developing epilepsy at some point in their lives. Temporal lobe epilepsy (TLE) is the most common form of focal epilepsy as medial structures, and the hippocampus in particular, are prone to generating seizures. Selective anterior temporal resection (which removes the hippocampus) is the most effective intractable TLE treatment, but given the critical role of the mesial temporal lobe in memory functioning, resection can have negative effects on this crucial cognitive skill. To minimize the adverse impact of temporal lobe surgery on memory functioning, reliable pre-surgical guides are needed. Clinical functional magnetic resonance imaging (fMRI) provides reliable, noninvasive guidance of language functioning and plays a growing role in the pre-surgical evaluation for epilepsy patients; however, localization of *memory* function in children with epilepsy using fMRI has not been established. Aside from the lack of neuroimaging memory studies in children with TLE, studies of typical development are limited. This review will focus on the functional anatomy of memory systems throughout development, with a focus on TLE. TLE provides the ideal model from which to understand memory function and the limits of plasticity and compensation/reorganization throughout development.

## Introduction

Memory is the process by which information is encoded, stored, and retrieved [1]. Memory can be divided into explicit (declarative) and implicit (nondeclarative) domains based on the manner information is stored and recalled [2]. Explicit memory encompasses factual knowledge (semantic memory) and memory for events and personal experiences (episodic memory) and involves at least four processes, including encoding, consolidation, storage, and retrieval. This review will focus on episodic memory, which is thought to develop through childhood. Both lesion and neuroimaging studies delineate the neural systems involved in episodic memory. Ghetti and Bunge [3] state that the development of episodic memory occurs through development of a brain network including the hippocampus, prefrontal cortex, and posterior parietal cortex.

Although episodic memory involves a distributed network, the medial temporal lobe (MTL), and specifically the hippocampus, has a particular role in episodic memory encoding and retrieval [4]. Many of the foundational lesion-based studies of memory were based on patients with temporal lobe epilepsy (TLE), and TLE provides a model to understand memory development. Thus, TLE will be a focus of the current review.

## Memory and epilepsy

### Adults

The MTL has a specific role in memory encoding and retrieval [4], yet this region is also prone to generating seizures, making TLE a common form of focal epilepsy [5]. Memory impairments are a common comorbidity in epilepsy [6–9]. Adults with TLE demonstrate material-specific memory deficits ipsilateral to seizure foci [6–9]—with left TLE associated with lower verbal memory on neuropsychological assessment and right TLE associated with lower visual memory [8, 9]. *Material specificity* describes each hippocampus’ processing of specific stimulus types (verbal—left; visual—right) and has been a hallmark concept in the neuropsychology of TLE. However, it is important to note that this theory may be oversimplified given the complexity of memory [10]. It has been posited that TLE patients with mesial temporal sclerosis (MTS) may show more material-specific deficits than those with more lateral TLE. Castro et al. [11] addressed this specific question and found that lateralization of verbal and nonverbal memory deficits was highly specific for left and right MTS (left MTS 82.2%, right MTS 92%) but infrequent (left MTS 11/43 or 25.6%, right 11/42 or 26.2%). Thus, there is evidence that material-specific memory deficits occur in TLE, particularly with MTS, but they do not occur ubiquitously.

Epilepsy surgery studies, however, provide further support for *material specificity.* The most effective intractable TLE treatment is selective anterior temporal resection [12, 13]; however, this can have negative effects on memory given the role of the MTL in memory functioning [14–18]. Postoperative decline in memory skills has been documented which correlates with the side of focus/surgical intervention [14–16, 19, 20], providing additional support for the *material specificity* hypothesis. Preoperative verbal memory performance (via neuropsychological assessment) predicts postoperative memory outcome for adults after left anterior temporal resections [6, 21].

### Children

Seventy percent of children and adolescents with epilepsy describe problems with learning and memory [22], and both parents and patients indicate that the cognitive effects of epilepsy are a primary concern [23]. For children, characterizing and understanding these impairments is important because learning and memory are essential skills for success in academic and everyday functioning [24], including adaptive functioning, quality of life, and eventual employment. Children with epilepsy exhibit deficits in learning and memory [25, 26]; however, the severity and pattern of impairment is not clear, with findings ranging from global memory disruption [27–29] to no deficits [30, 31]. For pediatric TLE, although material-specific memory deficits have also been found [29, 32], the majority of studies do not show the adult pattern of pre-surgical lateralizing memory impairments [8, 33, 34]. One study found that adolescents with TLE (along with adults), but not children, show laterality differences in memory performance [8]. This provides evidence that the lateralizing profile may be more evident with age. Instead of this lateralizing profile, there is evidence that children with TLE have memory functioning similar to typically developing (TD) controls [33] and that patients with mesial TLE have worse memory functioning than patients with lateral TLE [34]. Furthermore, regardless of side of focus, verbal memory may be most affected in pediatric TLE [35] and in pediatric epilepsy in general [36].

In surgical populations, overall, children with epilepsy do not show material-specific memory impairments postoperatively. A review reported that postoperative memory decline was noted in four out of 13 studies, while six out of 13 documented no change, and three out of 13 showed an improvement in memory functioning [37]. These results suggest children, unlike adults, are less reliant on the dominant MTL for memory functioning. However, Law et al. [38] compared temporal lobe resections with and without inclusion of the mesial structures (hippocampus and amygdala). They found that patients (age 5–19) with left mesial temporal lobe resections had declines in verbal memory, and thus, the authors suggest that previous studies did not analyze the surgical details when discussing postoperative memory decline. Further, children in this group were most at risk for verbal memory decline when they had (1) typical language lateralization (i.e., left dominant) and (2) good preoperative verbal memory (> 85 Standard Score). In sum, patients with left mesial temporal resections may have postoperative declines in verbal memory (but not to the extent observed in adults), while children with extra-mesial resections do not.

Overall, important differences exist between children and adults in epilepsy and the effects of temporal resection on memory networks. Children with TLE may not show the adult pattern of pre- or post-surgical lateralizing memory impairments, but there is some recent evidence that those with mesial temporal resections might. Neuroimaging may help to answer many of these questions regarding developmental differences in hippocampal functioning.

## Neuroimaging (fMRI) and memory

### Task-based fMRI

#### Typical development

**Fig. 1:**
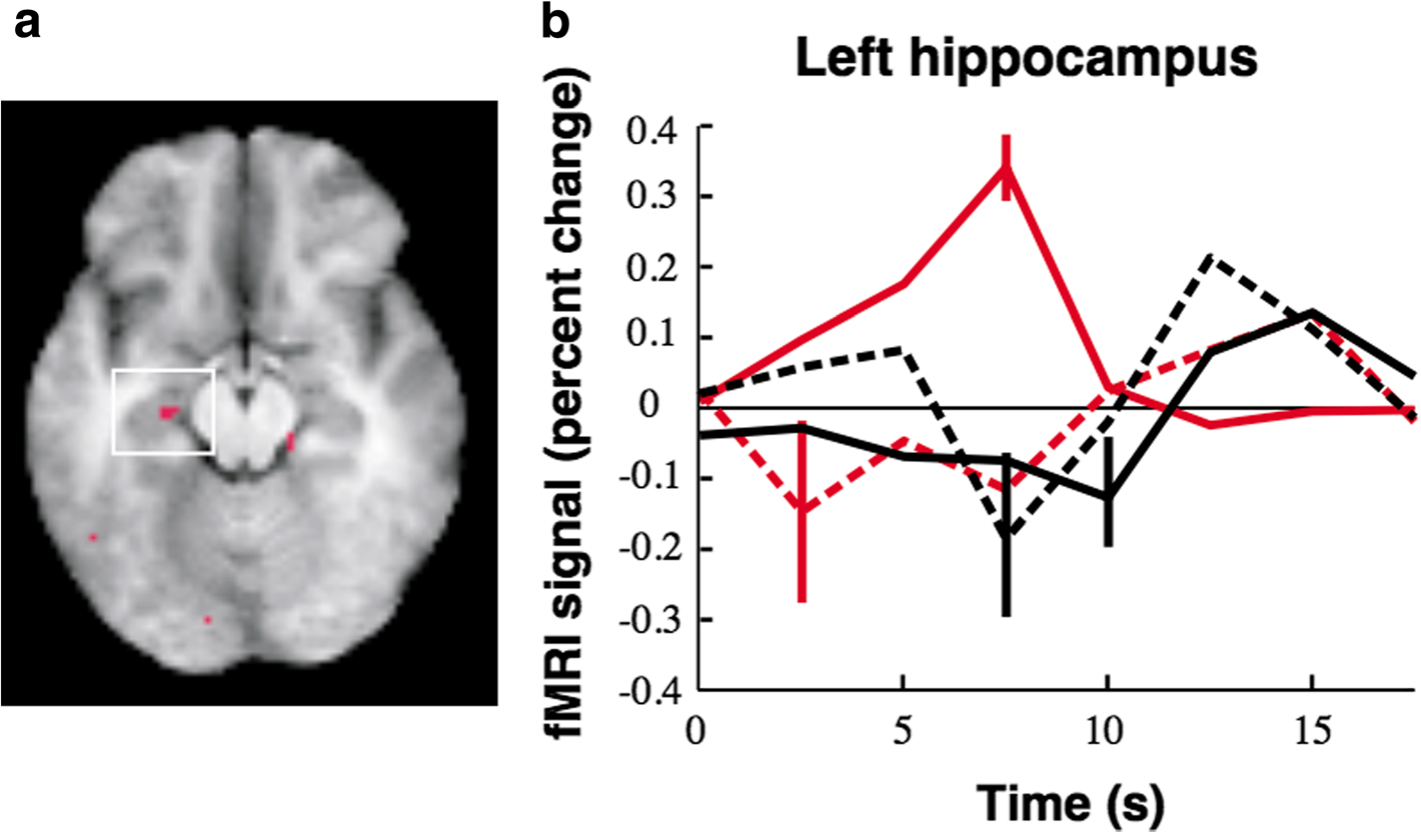
Results from hippocampal region of interest, comparing correct R and correct K response amplitudes (**a**). Event-related responses within the left hippocampal region; response amplitude for correct R trials was greater than that for correct K trials (**b**). Adapted with permission from Eldridge et al. [40]

**Fig. 2:**
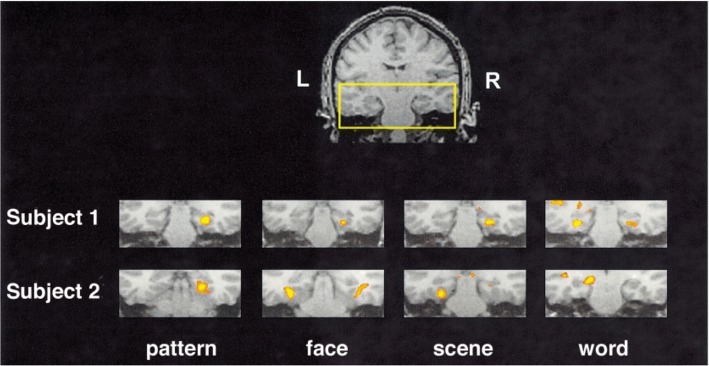
Activation in MTL for two participants. Pattern encoding yielded right, word yielded left, and face and scene encoding yielded bilateral activation. Adapted with permission from Golby et al. [96]

A complete review of memory fMRI functioning for adult TD populations is beyond the scope of the current review (for review, see Rugg and Vilberg, [39]). Rugg and Vilberg [39] review two of the main types of memory fMRI paradigms employed that dissociate recollection and familiarity. One uses the “Remember/Know” procedure, where participants report whether recognition of an item is accompanied (Remember) or unaccompanied (Know) by retrieval of contextual details during studying. The other requires a judgment about contextual features during studying (a “source memory” judgment, e.g., color of font). In sum, recollection-sensitive fMRI effects have been found in the hippocampus, parahippocampal, retrosplenial/posterior cingulate, lateral parietal cortices, and mPFC. There is evidence from adult TD studies that activity in the hippocampus increases when retrieval is associated with conscious recollection of a learning episode, and not when items are recognized only by familiarity or unrecognized [40] (see Fig. 1). Further, fMRI provides evidence for material specificity, such that each hippocampus is specialized to process specific types of information in TD adults; with verbal encoding resulting in left lateralization [41, 42] and visual encoding in right [41] (see Fig. 2).

Studies of memory functioning in TD children using fMRI are sparse (for review, see Ghetti and Bunge, [3]; see Table 1). The majority have focused on visual memory, memory for detail/complexity, or longitudinal hippocampal activation. Regarding visual memory, during encoding of visual scenes in children (age 11–19), one study found that MTL response decreased with age, while activation in the left dorsolateral PFC increased with age [14]. Similarly, Ofen et al. [43] studied adults and children (age 8–24) during encoding of visual scenes and found greater activation in subsequently remembered scenes than in forgotten scenes in the MTL and PFC areas and that the activation within the PFC increased with age. In a study of spatial memory, adults (age 18–25) demonstrated activation in the head and body of the hippocampus, but children (age 8–9 and 10–11) did not [44]. Differences between the age groups were also found in the posterior parietal cortex, anterior prefrontal cortex, and insula. These age-related differences in hippocampal activation are similar even in studies of verbal memory. A study of verbal memory in TD children (age 7–19) showed left hippocampal and basal ganglia activation for verbal memory in children, which declined as age increased [16].

Several studies have found differences in recollection for detail or complexity throughout development. Ghetti et al. [45] used an incidental encoding task to study groups of children across the age span (8-year-olds, 10–11-year-olds, 14-year-olds, and young adults) and found functional changes across development such that the hippocampus and parahippocampal gyrus became increasingly more specialized for detailed recollection. Chai et al. [46] examined indoor and outdoor scenes of varying complexity in children and young adults (age 8–24) and found that recognition memory improved with age for high-, but not low-complexity scenes [46]. The authors concluded that the right posterior parahippocampal gyrus is important for the development of long-term recollection for high-complexity scenes. Similarly, Maril et al. [47] studied children (age 8–11) and young adults with an fMRI paradigm consisting of noun/color combinations. The adults recruited left PFC and parietal and occipital-temporal cortices, while the children recruited the right occipital cortex, providing evidence that episodic encoding for children depends more on perceptual systems, whereas top-down frontal control and parietal structures are involved more with age.

The complexity of understanding memory is also in part due to the complexity of the hippocampus. The hippocampus can be divided longitudinally (anterior, body, posterior) and by subregions (CA fields, dentate gyrus). The complexity of the subregions of the hippocampus is an important variable to be considered and an area of current focus in the field, which is beyond the scope of this review. Some studies segmenting the hippocampus longitudinally will be briefly reviewed here. In typical development, structural and functional developmental changes occur along the longitudinal axis of the hippocampus. Structurally, decreased anterior and increased posterior hippocampus volume occurs from age 4 to 25 (more pronounced on left) [48]. Another study found a positive relationship between episodic memory and anterior hippocampal volume in both the left and right hemispheres for 6-year-old children but not for 4-year-old children, indicating varying, dynamic changes in anterior and posterior volume throughout development [49]. Functionally, episodic recall is associated with activity in the posterior hippocampus in TD children and anteriorly in TD adults [50]. A more recent study by Sastre et al. [51] found that good memory performance in adults was associated with more focal hippocampal recruitment (hippocampal head) compared to high-performing 10–11-year-olds and low-performing adults where activation was observed across the entire hippocampus. Thus, even within this hippocampal structure, there are subcomponents that may have functional differences over development.

#### Epilepsy

For both children and adults with epilepsy, reliable pre-surgical guides are necessary to predict and minimize the adverse impact of temporal resection on memory functioning. Neuropsychological assessment and the intracarotid amobarbital procedure (Wada) can be used for this purpose, but the former is not highly predictive of postoperative outcome in children [37] and the latter is invasive and challenging to perform in children. Transition to a reliable, non-invasive procedure, as in language mapping, reduces risk for morbidity and extends the lower age limit for studies. fMRI is one promising alternative for assessing memory functioning pre-surgically; however, memory paradigms have been difficult to use clinically. One challenge is designing paradigms that increase cerebral blood flow sufficiently to detect MTL/hippocampal signal on an individual basis because memory processing pathways are always “online” [52]. Another challenge is that the hippocampus is difficult to image due to location-based vulnerability to susceptibility artifacts. Despite these difficulties, many group-level fMRI memory studies have been conducted in adults with epilepsy (see Table 2) [14, 20, 41, 53–68]. Few of these studies have employed the paradigms common in TD adults, such as the Remember/Know paradigms (for review, see Rugg and Vilberg [39]). This is likely due to the complexity of these paradigms, which can be challenging for patients with any cognitive difficulties. Further, the majority of epilepsy studies have only conducted fMRI during memory encoding, with recognition or recall testing after scanning (see Table 2).

**Table 1:** fMRI memory studies for TD children

Author/year	Population	fMRI paradigm: verbal or visual	Recall tested during scan or post scan
Menon et al. 2005 [97]	11–19 (*n* = 25)	Visual: scenes	Post scan
Ofen et al. 2007 [43]	8–24 (*n* = 49)	Visual: scenes	Post scan
Chai et al. 2010 [46]	8–24 (*n* = 52)	Visual: scenes (varying complexity)	Post scan
Ghetti et al. 2010 [45]	8, 10–11, 14, and young adults (*n* = 80, 20 in each group)	Visual: drawings (incidental encoding task)	Post scan
Maril et al. 2010 [98]	7–19 (*n* = 24)	Verbal: words (incidental encoding task)	Post scan
Maril et al. 2011 [47]	8–11, young adults (*n* = 33)	Both: noun/color combinations	Post scan
Demaster et al. 2013 [44]	8–9, 10–11, 18–25 (*n* = 48)	Visual: spatial memory task	During scan
Demaster & Ghetti 2013 [50]	8–11, 18–25 (*n* = 41)	Visual: drawings	During scan
Sastre et al. 2016 [51]	8–9, 10–11, adults (*n* = 126)	Visual: item-scene pairs	During scan

Summary of several published studies of memory fMRI for TD children in chronological order, including study population, fMRI tasks, and whether recall was tested during scanning

*TD* typically developing

Several of these studies show that some adults with TLE demonstrate greater activation in MTL contralateral to the seizure focus (e.g., for left TLE, verbal encoding yields activation in right MTL) [41, 42, 69]. Memory fMRI can also predict postoperative memory outcomes for adults with TLE. Models of adult postoperative functioning propose that these outcomes are predicted either by activation in the hippocampus to be resected (*hippocampal adequacy*) or the non-resected hippocampus (*hippocampal reserve*) [58]. Adults with refractory TLE demonstrate greater memory decline following temporal resection with greater ipsilateral compared to contralateral MTL activation on preoperative fMRI memory, supporting *hippocampal adequacy* [20, 56, 59, 62, 70]. Research also shows that greater pre- than postoperative ipsilateral posterior MTL activation is related to better verbal memory after left anterior temporal lobe resection [67]. These results provide evidence that the capacity of the posterior remnant of the ipsilateral hippocampus is important for postoperative verbal memory functioning, not the functional reserve of the contralateral hippocampus or early postoperative reorganization to ipsilateral posterior MTL.

Most of the fMRI memory studies that have been conducted in adults with epilepsy are group-level studies (see Table 2) [14, 20, 41, 53–68]. Two of those studies have also shown that memory fMRI paradigms can predict postoperative memory outcome with *individual* fMRI results [61, 68]. Frings et al. [61] studied 22 patients with mesial TLE using a spatial memory task in a 3D virtual environment. They found that hippocampal activation lateralization during this task was significantly correlated with postoperative decline in verbal memory, such that greater activation of the to-be-resected hippocampus was related to increased verbal memory decline. Sidhu et al. [68] studied 50 patients with TLE and 26 controls with an fMRI memory word list encoding paradigm. Results showed that left lateralization within a medial temporal and frontal mask could predict verbal memory postsurgical outcome for individual patients, while bilateral posterior hippocampal activation correlated with less verbal memory decline.

**Fig. 3:**
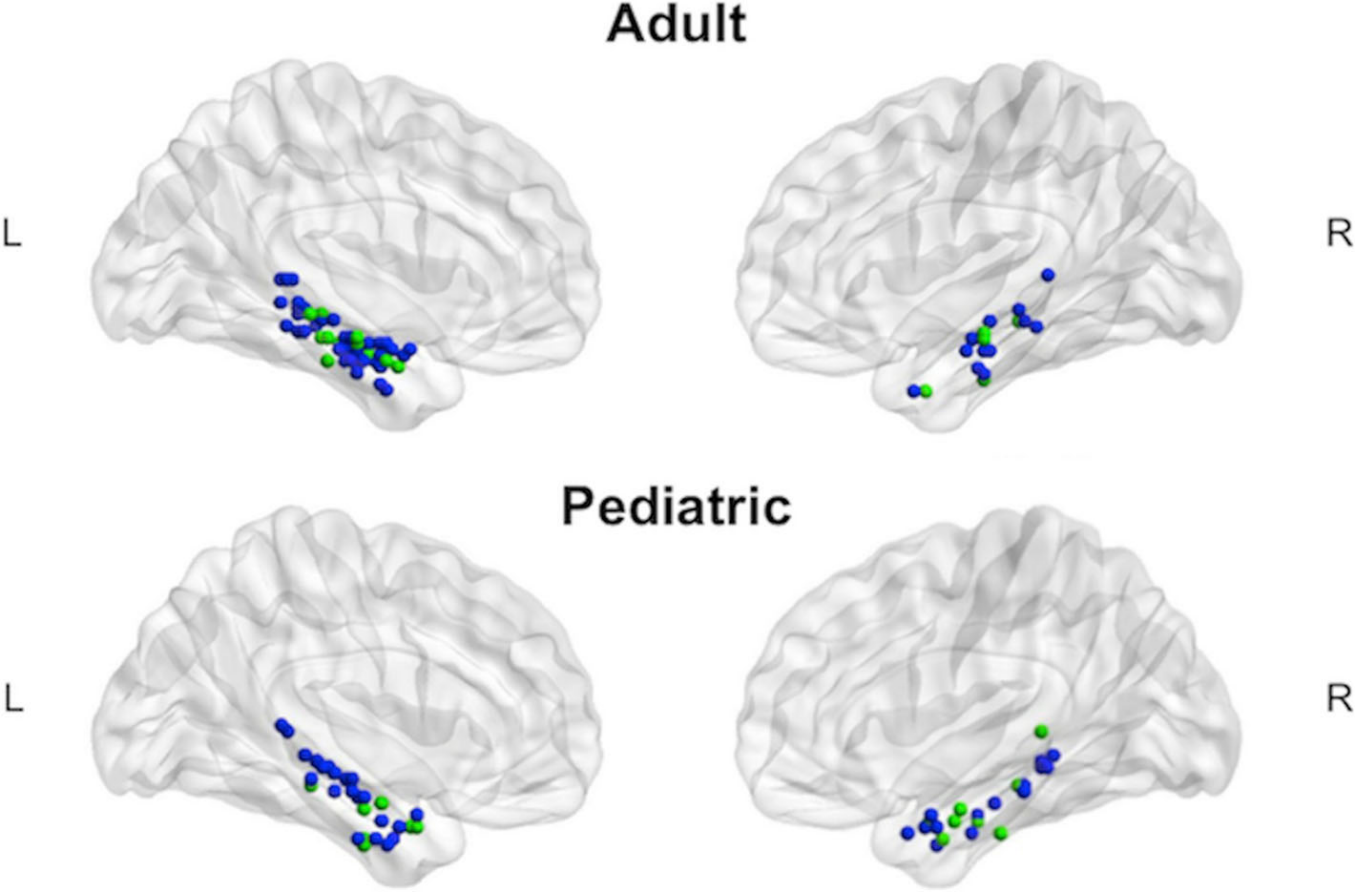
During language fMRI, the peak MTL voxel is depicted for each individual in the adult and pediatric age group (blue represents TD controls and green represents epilepsy). MTL was more left lateralized in adults than children. MTL, mesial temporal lobe. Adapted with permission from Sepeta et al. [71]

As a result of this work, recent AAN Practice Guidelines indicate moderate (Level B) evidence for memory fMRI. However, to our knowledge, no prior fMRI studies of memory functioning in pediatric TLE have been published; thus, this is an important area of future investigation. In Sepeta et al. [71], we measured hippocampal activation indirectly via a language fMRI task in TD children and adults, as well as those with focal epilepsy (see Fig. 3). We found a developmental difference in MTL lateralization such that MTL activation was left lateralized for adults, but less so in children. Each individual’s peak voxel within the bilateral MTL ROIs illustrates that adults have more left-lateralized activation than children. Language lateralization (Broca’s and Wernicke’s) predicted 19% of the variance in MTL lateralization for adults, but not for children. These results suggested a developmental shift in lateralization of MTL function, with increased left lateralization across the age span. This shift may help explain why children have better memory outcomes following resection compared to adults. Children may initially engage both MTLs, with specialization for specific types of material (verbal—left MTL, visual—right MTL) occurring later in development. This follows research demonstrating increased left lateralization for language [72] and right lateralization for visuospatial functions with age [73]. For children with epilepsy who undergo surgery, these cognitive functions may be preserved due to persistence of (earlier) developmental processing capacity in contralateral homologs (Developmental Origins Hypothesis).

**Table 2:** fMRI memory studies for adults with epilepsy

Author/year	Population	fMRI paradigm: verbal or visual	Recognition or Recall tested during scan or post scan
Detre et al. 1998 [53]	10 TLE, 8 TD	Visual: scenes	Post scan
Killgore et al. 1999 [54]	9 patients who underwent ATL	Visual: scenes	Post scan
Dupont et al. 2000 [55]	7 left MTLE (MTS), 10 TD	Verbal: supra-span list of abstract words	During scan (silently recall) and post scan
Jokeit et al. 2001 [56]	30 TLE, 17 TD	Visual: Roland’s Hometown Walking task	During scan
Golby et al. 2002 [41]	9 MTLE	Both: 4 tasks: patterns, faces, scenes, words	Post scan
Richardson et al. 2003 [57]	24 LMTS, 12 TD	Single words	Post scan
Rabin et al. 2004 [58]	35 TLE, 30 TD	Visual: scenes	Post scan
Janszky et al. 2005 [17]	16 MTLE	Visual: Roland’s Hometown Walking task	During scan
Richardson et al. 2006 [59]	30 MTLE (MTS), 13 TD	Single words (same as Richardson et al. 2003 [57])	Post scan
Avila et al. 2006 [60]	25 with lesions in the temporal lobe, 12 TD	Visual: 2 tasks: (1) picture encoding and (2) Roland’s Hometown Walking task	(1) Picture encoding = post scan, (2) Roland’s Hometown Walking task = during scan
Powell et al. 2007 [62]	15 TLE	Both: 1 task with pictures, faces, words	Post scan
Frings et al. 2008^a^ [61]	22 MTLE	Visual: spatial memory task (3D virtual environment)	During scan (recognition task)
Binder et al. 2010 [63]	30 patients who underwent LATL, 37 RATL	Visual: scenes	Post scan
Dupont et al. 2010 [64]	25 MTLE	Visual: pictures of objects (learned outside of scanner and fMRI 24 h later)	During scan (recognition task)
Bonelli et al. 2010 [14]	72 TLE, 20 TD	Both: 1 task with pictures, faces, words (same as Powell et al. 2007 [62])	Post scan
Alessio et al. 2013 [65]	17 MTLE, 9 TD	Both: 1 task with abstract designs and abstract words	During scan (silently recall)
Bonelli et al. 2013 [67]	46 TLE	Both: 1 task with pictures, faces, words (same as Powell et al. 2007 [62] and Bonelli et al. 2010 [14])	Post scan
Sidhu et al. 2013 [66]	44 MTLE, 26 TD	Both: 1 task with faces, words	Post scan
Sidhu et al. 2015^a^ [68]	50 MTLE, 26 TD	Verbal: words	Post scan

Summary of several published studies of memory fMRI for adults with epilepsy in chronological order, including study population, fMRI tasks, and whether recall was tested during scanning

*TLE* temporal lobe epilepsy, *MTLE* mesial temporal lobe epilepsy, *MTS* mesial temporal sclerosis, *TD* typically developing

^a^Includes individual-level results

### Resting-state connectivity

#### Typical development

Resting-state connectivity involves a “task-free” MRI paradigm while patients are at rest, while remaining awake and alert. Resting-state fMRI does not require specific tasks to assess for different functions (e.g., motor, language), and many networks can be tested simultaneously, saving scanning time. It is less demanding than task-based fMRI and thus can be used in patients with neurological and cognitive deficits, who might be precluded from participating in task-based fMRI. Resting-state has been found to be reliable across imaging sessions [74, 75] and different study participants [75, 76]. However, there are some disadvantages, which include that the physiological basis of the resting-state signal is not completely understood and the state is unmonitored; therefore, information about the cognitive processes during resting state (e.g., inner speech) is not available (for review, see Goodyear et al. [77]).

Memory involves a distributed neuroanatomical network, and hippocampal connectivity to this network is an indicator of episodic memory function. In TD adult resting-state functional connectivity (FC) studies, a memory network has been identified between the hippocampus and medial PFC, lateral temporal and several parietal regions (including posterior cingulate cortex (PCC), retrosplenial cortex, and bilateral inferior parietal lobule (IPL)) [78]. Specific regions in this network are associated with successful memory recall, such as the PFC, IPL, and the medial surface extending from the retrosplenial cortex to PCC.

In typical development, many resting-state networks have been identified as early as 26 weeks gestation [79]. Resting-state studies of typical development in early and middle childhood have revealed network patterns common in children [80–83]. Many of the resting-state networks in TD children are similar to adults, but consistent differences include decreased long-range and increased short-range correlations [84]. A recent study of typical development in 4- and 6-year-olds found similar hippocampal FC to adults [85]. The regions connected to the hippocampus included the lateral temporal regions, precuneus, and multiple parietal and prefrontal regions. The strength of posterior hippocampal FC with the right middle temporal gyrus was associated with memory ability. Another study of children aged 4–10 found an increase over age in strength of hippocampal connectivity with lateral temporal lobes and the anterior cingulate [86]. Age-controlled analyses showed similar hippocampal–parietal memory network identified in adults.

#### Epilepsy

For adults with TLE, several resting-state studies have shown mixed results. Some find a reduction in hippocampal FC either ipsilateral to the seizure focus [69, 87] or bilaterally [88, 89] in adults with TLE. Alternatively, some find strong ipsilateral hippocampal FC [90]. Haneef et al. [91] found both increased and decreased hippocampal FC, with increased FC to the limbic network (temporal lobe, insula, thalamus), frontal and angular gyri, basal ganglia, brainstem, and cerebellum, and reduced hippocampal FC with the sensorimotor cortex and default mode network. Overall, the left TLE group often shows more marked FC changes than right TLE [92].

Hippocampal FC during resting state has not been studied systematically with a pediatric TLE population. Some studies of FC in children with *focal* epilepsy have been conducted using resting-state fMRI. Vaessen et al. [93] found that children with frontal lobe epilepsy demonstrated higher modularity than controls, indicating that subnetworks were underconnected in epilepsy. Overall, patients demonstrated abnormal modular organization, with a decrease in long-range and increase in interhemispheric connectivity in patients. Cognition (performance and reaction time on a computerized visual searching task) was associated with higher modularity scores for patients. Besseling et al. [94] studied children with Rolandic epilepsy and found reduced FC between the left sensorimotor area and right inferior frontal gyrus in patients. FC between those two regions was positively correlated with language scores for the patients.

In sum, resting-state fMRI is a promising method for patients with neurological and cognitive deficits. For TLE, many resting-state studies have focused on hippocampal FC with adults and, these have shown mixed findings; however, more research is necessary for both typical development and pediatric TLE.

## Conclusion

In summary, investigation of memory functioning with advanced neuroimaging has implications for developmental neuroscience, as well as clinical practice. Our understanding of the basic neuroscience concept of neural plasticity has already benefited from studies of language reorganization in epilepsy, showing atypical language lateralization is more prevalent in patients with early seizure onset [95]. However, little is known about processes facilitating memory reorganization, and there is controversy about the degree to which function is preserved or impaired due to reorganization. Aside from the lack of neuroimaging memory studies in children with TLE, studies in TD children are limited. Important differences exist between children and adults in epilepsy and the effects of temporal resection on memory networks, suggesting a specific window for memory plasticity. It is plausible that children use both hippocampi in memory encoding and retrieval, and that across typical development hippocampi become material-specific. If true, this would have a significant impact on our understanding of *material specificity* in memory, as well as epilepsy interventions, such as surgical resection. More neuroimaging studies of memory are necessary, as studies of typical development are limited and there is a lack of neuroimaging memory studies in children with TLE. Resting-state studies can provide meaningful information regarding the development of memory systems, but more research is also required using this technique. Further study of memory via both task-based and resting-state fMRI for TD and children with TLE is crucial to noninvasively ascertain the developmental trajectory of postoperative risk for memory decline.
